# The United States Veterinary Medical Association

**Published:** 1888-01

**Authors:** 


### THE UNITED STATES VETERINARY MEDICAL ASSOCIATION.

We regret the loss, through fault of the printer, of the manuscript prepared for the October number of the JOURNAL, giving report and criticism of the twenty-fourth annual meeting of this Association. The report will be found in another part of the present number ; it is to be hoped that future reports may be enabled to contain more than the meagre noting of a day occupied by discussion of minor technicalities, while the majority of the members wait in vain for the subject-matter.

Intelligence and education, with a uniform standard of of the latter must be the result of the activity of the members of the Association, in making their own examples, those to be emulated by the coming generation rather than the result of the generalities of committee reports. The new President was, unexpectedly to himself, called to assume the chair between the sessions of the meeting, and unaware of the foundation of the Prize Committee by the Constitution, allowed a reconsideration of the very proper report of the Chairman of this Committee. By a large vote the members awarded the prize to the paper on "Schirrus Cord;" upon examination of the Constitution we find this award to have been unconstitutional. We would call the attention of the Editor of the *Review* to the notice given by the

President before action was taken, that any reconsideration of the subject could only be upon the prize offered by the Association.

The Semi-Annual meeting of the Association will be held in Baltimore on March 20th, 1888. A plain duty lies before the members—the preparation of subjects of interest in the advancement of Veterinary Medicine and attendance at the meeting in a spirit proper to discuss these subjects from a scientific standpoint, so that the results will be of benefit to others as well as to themselves. H.

